# Supplementary figures and images for: Effects of origin, seasons and storage under different temperatures on germination of Senecio vulgaris (Asteraceae) seeds
Source: PeerJ. 2016 Aug 17;4:e2346. doi: 10.7717/peerj.2346 (PMC4991864; doi:10.7717/peerj.2346)

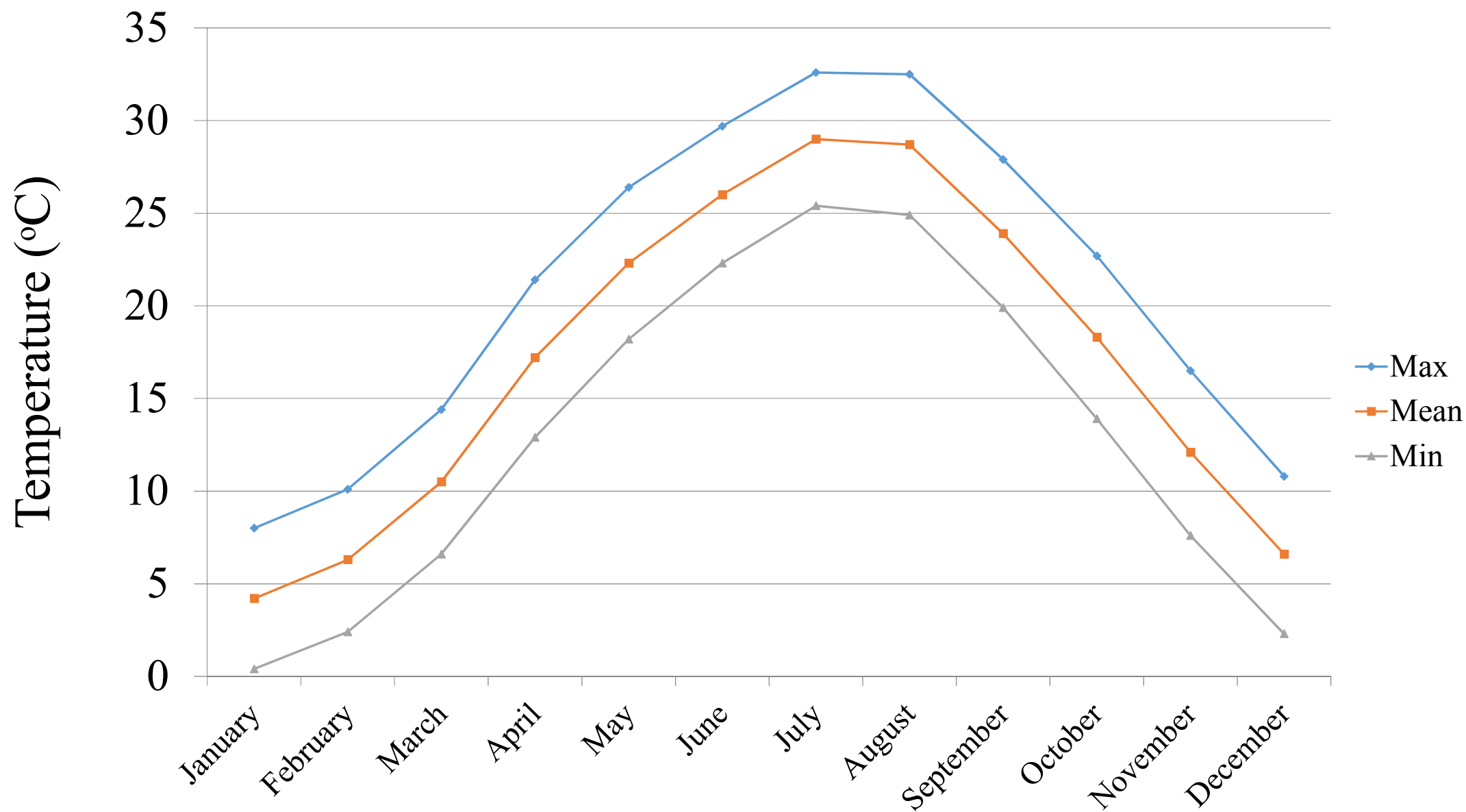

Supplement: Figure S1 [file peerj-04-2346-s002.pdf]
