# Supplementary material for: Effects of origin, seasons and storage under different temperatures on germination of Senecio vulgaris (Asteraceae) seeds
Source: PeerJ. 2016 Aug 17;4:e2346. doi: 10.7717/peerj.2346 (PMC4991864; doi:10.7717/peerj.2346)
Supplement: Table S1 — The results of the Kruskal-Wallis test shown the difference of Germination Percentage (GP) and Mean germination time (MGT) between populations of Senecio vulgaris. Outdoor germination experiments were conducted in autumn, summer and by using seeds stored at different conditions. For all 8 tests, df = 11, N = 36. [file peerj-04-2346-s003.docx]

| season | storage | log（GP） | | log（MGT） | |
| --- | --- | --- | --- | --- | --- |
|  |  | x^2^ | P | x^2^ | P |
| Autumn | 4℃ | 14.9 | 0.18 | 9.08 | 0.61 |
|  | Lab temperature (ca. 27℃) | 20.8 | 0.035 * | 18.57 | 0.07 |
| Summer | 4℃ | 7.43 | 0.76 | 7.43 | 0.76 |
|  | Lab temperature (ca. 27℃) | 14.29 | 0.22 | 16.76 | 0.12 |
